# Structural, Electrical, and Optical Properties of Single-Walled Carbon Nanotubes Synthesized through Floating Catalyst Chemical Vapor Deposition

**DOI:** 10.3390/nano14110965

**Published:** 2024-06-02

**Authors:** Melorina Dolafi Rezaee, Biplav Dahal, John Watt, Mahir Abrar, Deidra R. Hodges, Wenzhi Li

**Affiliations:** 1 Department of Physics, Florida International University, Miami, FL 33199, USA; mdola008@fiu.edu (M.D.R.); bdaha002@fiu.edu (B.D.); 2 Center for Integrated Nanotechnologies, Los Alamos National Laboratory, Los Alamos, NM 87545, USA; watt@lanl.gov; 3 Department of Electrical & Computer Engineering, Florida International University, Miami, FL 33174, USA; mabrar@fiu.edu (M.A.); dhodges@fiu.edu (D.R.H.)

**Keywords:** SWCNTs, FCCVD method, Raman spectroscopy, Hall effect measurements, acid treatments, FTIR

## Abstract

Single-walled carbon nanotube (SWCNT) thin films were synthesized by using a floating catalyst chemical vapor deposition (FCCVD) method with a low flow rate (200 sccm) of mixed gases (Ar and H_2_). SWCNT thin films with different thicknesses can be prepared by controlling the collection time of the SWCNTs on membrane filters. Transmission electron microscopy (TEM) showed that the SWCNTs formed bundles and that they had an average diameter of 1.46 nm. The Raman spectra of the SWCNT films suggested that the synthesized SWCNTs were very well crystallized. Although the electrical properties of SWCNTs have been widely studied so far, the Hall effect of SWCNTs has not been fully studied to explore the electrical characteristics of SWCNT thin films. In this research, Hall effect measurements have been performed to investigate the important electrical characteristics of SWCNTs, such as their carrier mobility, carrier density, Hall coefficient, conductivity, and sheet resistance. The samples with transmittance between 95 and 43% showed a high carrier density of 10^21^–10^23^ cm^−3^. The SWCNTs were also treated using Brønsted acids (HCl, HNO_3_, H_2_SO_4_) to enhance their electrical properties. After the acid treatments, the samples maintained their p-type nature. The carrier mobility and conductivity increased, and the sheet resistance decreased for all treated samples. The highest mobility of 1.5 cm^2^/Vs was obtained with the sulfuric acid treatment at 80 °C, while the highest conductivity (30,720 S/m) and lowest sheet resistance (43 ohm/square) were achieved with the nitric acid treatment at room temperature. Different functional groups were identified in our synthesized SWCNTs before and after the acid treatments using Fourier-Transform Infrared Spectroscopy (FTIR).

## 1. Introduction

Carbon nanotubes (CNTs) are cylindrical molecules made of rolled-up sheets of single-layer carbon atoms (graphene) [1]. SWCNTs have diameters in the range of 1–2 nanometers (nm), double-walled carbon nanotubes (DWCNTs) have diameters in the range of 2–4 nm, and multi-walled carbon nanotubes (MWCNTs) have diameters in the range of a few nanometers to one hundred nanometers. MWCNTs consist of many concentrically stacked nanotubes with lengths of several micrometers or millimeters [2]. The credit for discovering MWCNTs and SWCNTs is given to Ijima [3,4] in 1991 and 1993, respectively.

Due to their lightweight and one-dimensional structure, SWCNTs show unique mechanical, electrical, optical, and thermal properties [5,6,7]. For example, their electrical conductivity is estimated theoretically to be 10^8^ S/m, and they exhibit a high thermal conductivity of 3500 W/k.m. The theoretical tensile strength of individual SWCNTs is around 100 Gpa, with a Young’s modulus of 1 TPa [8,9,10]. Because of these distinctive properties, SWCNTs can be utilized in a wide range of applications, such as flexible and transparent microelectronics, energy storage and conversion devices, multifunctional composites, aerospace devices, electric conductors, etc. [11,12].

Compared to other methods like arc discharge or laser ablation, chemical vapor deposition (CVD) is one of the techniques most used to synthesize SWCNTs due to its high yield, low impurity, and gentle synthesis conditions [13]. So far, different CVD processes have been introduced to fabricate SWCNTs, such as aerosol-assisted CVD [14], microwave-plasma-enhanced CVD [15], hot-filament CVD [16], oxygen-assisted CVD [17], FCCVD, etc. Compared to the other CVD techniques, FCCVD is more appropriate for the mass production of SWCNTs because of its low cost, good flexibility, scalability, and controllability [18,19]. The properties of SWCNT films strongly depend on the morphology of the SWCNTs’ bundles, like their length, diameter, density, etc. [20], which can be efficiently controlled in the FCCVD technique.

In the FCCVD method, the appropriate choice of growth parameters plays an important role in controlling the growth of SWCNTs. Thiophene is commonly used to deliver the sulfur components used in SWCNT synthesis [21]. Sulfur plays a vital role in nucleation, renucleation, surface chemistry, and the aerogel formation of the catalyst nanoparticles. On the other hand, the catalyst affects the SWCNTs’ morphology and helps the growth of the SWCNTs by reducing the synthesis energy [22]. Because of the low bonding energy between the sulfur and carbon atoms, thiophene decomposes earlier than ferrocene (catalyst) in the initial phase of the FCCVD reaction. The results of this process are hydrocarbon species and liberated sulfur atoms. A thin layer of coating is formed on the surface of the metallic catalyst by these liberated sulfur atoms. This is advantageous to the growth of SWCNTs because the sulfur surfactant can prevent the encapsulation of carbon particles. Moreover, it can stop agglomeration between the sulfur-coated catalyst particles [23].

The type, morphology, and crystallinity of the synthesized SWCNTs can be determined by carbon precursors such as ethanol. The carbon precursor is dissociated after being absorbed on the surface of the catalyst. A SWCNT cap is formed by the transformation of a closed carbon network. The SWCNT growth continues by generating carbon precipitation from the catalyst below the SWCNT cap. The growth stops after the termination of the catalysts [24] by the complete sulfur coverage on the surface of the catalyst particle [22,23]. SWCNTs’ diameter distribution is known to be affected by the composition of carrier gases. As an example, it was observed that increasing Ar as a carrier gas leads to a decrease in SWCNTs’ diameter distributions [25]. A small diameter distribution is favorable for the reproductivity and consistency of SWCNT products.

Hydrogen is commonly used as a carrier gas in the FCCVD system. This gas is found to be very beneficial to the synthesis of SWCNTs. It can help the breakdown of the hydrocarbon precursors [26] and preserve the catalyst’s lifetime [27]. Moreover, hydrogen induction can effectively remove amorphous carbon, leading to an improvement in CNT synthesis [28].

Controlling the electronic properties of SWCNTs can develop their technical applications in different fields. SWCNTs can show either semiconducting or metallic behaviors depending on their chiral vector. The electronic properties of SWCNTs can be improved by doping using acid treatments. Brønsted acids (HCL, HNO_3_, H_2_SO_4_, …) have been known to have electrochemical effects on graphite, leading to acceptor doping [29,30]. The use of SWCNT thin films, composed of either a random or oriented network, is an incredibly promising technology for the future of electronics. SWCNT films with both high electrical conductivity and optical transparency can serve as transparent electrodes in place of conventional indium tin oxide (ITO). Studies have demonstrated that SWCNTs possess high electronic mobilities ranging from 10,000 to 100,000 cm^2^/V s, and high conductivities up to 400,000 S/cm. However, it is still a challenge to extend the exceptional electrical properties of individual SWCNTs to two-dimensional networks. Without chemical doping, high-quality SWCNT films show sheet resistances of 300–1000 ohm/square at around 85% optical transmittance, which are significantly higher than ITO’s 10–20 ohm/square at 90% transmittance [31]. Transferring charges (electrons or holes) to the nanotube through intercalation and/or functionalization processes is one way to change the carbon nanotube’s electronic and vibrational characteristics [32,33,34]. Therefore, it is important to study the effect of doping on SWCNTs’ electrical properties.

In this work, we report the production of clean SWCNT films using the FCCVD method. We followed a process similar to that used by Zhang et al. [35] to synthesize SWCNTs, with some changes in the type of carrier gases, their total flow rate, and the synthesis procedure. Our synthesis process, with a total carrier gas flow rate of 200 sccm, can be considered a low-cost procedure. In this research, thiophene (C_4_H_4_S) was used as a growth promoter, ferrocene as the catalyst, ethanol as the carbon precursor, and argon and hydrogen as the carrier gases. The morphology and characteristics of the thin films were examined through scanning electron microscopy (SEM) and transmission electron microscopy (TEM). Raman spectroscopy was used to show the quality and crystallinity of the SWCNT films. The transmittance of the synthesized SWCNT films was obtained for the thin film samples with collection times of 5, 10, 15, 20, and 25 min, and the electronic properties of the synthesized SWCNTs were investigated using Hall effect measurements. The SWCNT films showed a high carrier concentration of 10^21^–10^23^ cm^−3^, which is in good agreement with the theoretical prediction [36,37]. H_2_SO_4_, HCL, and HNO_3_ were used for the treatment of the thin films, and the effect of different acid treatments on the mobility, conductivity, and sheet resistance of samples was studied. Fourier-Transform Infrared Spectroscopy (FTIR) was employed to determine the functional groups in the SWCNT films before and after the acid treatment.

Generally, SWCNTs are being actively researched and applied in various fields such as biomedicine, energy storage, and electronic devices. The p-type nature of our synthesized SWCNTs makes them suitable for use as a hole-transporting layer (HTL) in optoelectronic devices such as solar cells. The mobility of our pristine SWCNTs is in the order of 10^−2^ cm^2^/Vs, and after the acid treatment, it reaches the highest value of 1.5 cm^2^/Vs. This value is much higher than the reported value for the mobility of the spiro-OMeTAD (in the range of 8 × 10^−5^ to 2 × 10^−4^ cm^2^/Vs) [38], which is one of the widely used HTLs in perovskite solar cells (PSCs). Moreover, employing SWCNTs as the hole-transporting layer in PSCs can dramatically increase the stability of the cells because they can act as a barrier to air and moisture. Transparent conducting films (TCFs) are another potential application for our synthesized SWCNTs, especially after the acid treatments; this is due to a lower sheet resistance being reached at a higher transmittance. SWCNT-based TCFs can be employed in the touch screen, organic light-emitting diode (OLED), flat panel display, and thin-film solar industries [39].

## 2. Experimental

### 2.1. Materials

Ferrocene (98%, Sigma-Aldrich, St. Louis, MO, USA) was used as a catalyst precursor. Thiophene (99+%, Acros Organics, Thermo Fisher Scientific, Fair Lawn, NJ, USA) and ethanol (89–91%, Fisher Chemical, Fisher Scientific, Fair Lawn, NJ, USA) were used as the growth promoter and the carbon source, respectively. Argon (AR UHP300, Airgas, Radnor, PA, USA) and hydrogen (HY UHP300, Airgas) were employed as carrier gases. Millipore Express membrane filters (0.45 μm PES Membrane) were used to collect the SWCNTs. The SWCNT thin films were doped using sulfuric acid (H_2_SO_4_, 96.1 *w*/*w* %, Fisher Scientific, Fair Lawn, NJ, USA), nitric acid (HNO_3_, 69.4 *w*/*w* %, Fisher Scientific), and hydrochloric acid (HCL, 37.2 *w*/*w* %, Fisher Scientific).

### 2.2. SWCNTs Synthesis

**Figure 1:**
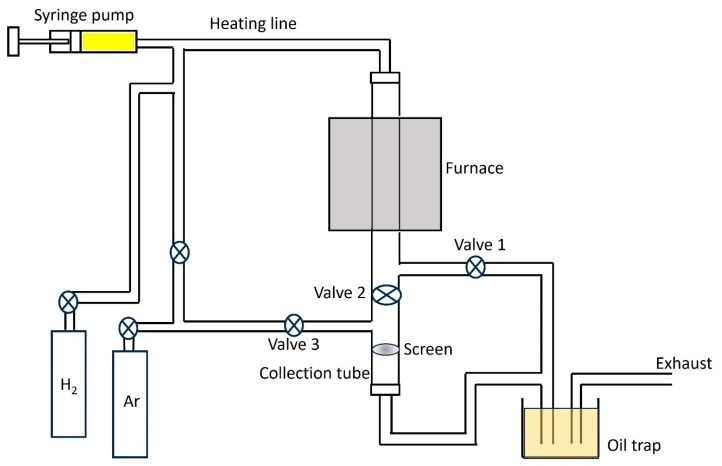
Schematic illustration of the FCCVD experimental setup for the SWCNT synthesis.

The precursor solution was prepared by dissolving ferrocene (0.4 wt %) and thiophene (molar ratio of S/Fe = 0.3) in 10 milliliters of ethanol. A syringe pump was used to inject the precursor solution into a heating line kept at 140 °C. The feeding rate of the syringe pump was adjusted at 6 μL/min. The precursor solution was evaporated in the heating line and carried into a vertical furnace by argon and hydrogen gases, each with a flow rate of 100 standard cubic centimeters (sccm). The temperature of the furnace was kept at 1000 °C during the experiment. During the growth time (30 min), valve1 was kept open while valve 2 and 3 were closed, so residual air in the reaction chamber and any materials initially produced during this time were exhausted through the oil trap (Figure 1). After that, valve 2 was opened and valve 1 was simultaneously closed to collect the pure and clean SWCNTs on a membrane filter, which was kept in a collection tube at room temperature. To increase the mass production of the CNTs, we added an extra valve (valve 3) (Figure 1). The SWCNT films were removed from the collection tube after being purged with argon at 100 sccm, and a new membrane filter was installed and purged with argon to get rid of the air inside the collection tube. Then, valve 2 was opened and valves 1 and 3 closed to collect the SWCNTs again. This procedure was repeated several times without stopping the experiment to produce several CNT thin films in one experiment.

### 2.3. SWCNT Characterization

The overall morphology of the CNT networks and bundles was characterized using scanning electron microscopy (SEM FS100, JEOL-F100, JEOL Ltd., Tokyo, Japan) under 1 KV. The energy-dispersive X-ray spectroscopy (EDS) technique was used to determine the elemental composition of the samples. Transmission electron microscopy (TEM), performed on a monochromated and aberration-corrected FEI Titan operating at 300 keV, was used to analyze the crystallinity of the CNTs, and ImageJ software (free online version 1.53t) was employed to determine the distance between the fringes in the bundles. Raman spectroscopy (633.8 nm He-Ne Laser, 514 nm Argon ion laser, and 785 nm diode laser) was used to obtain the Raman spectra of the SWCNTs. A UV–visible spectroscopy system (Hewlett Packard 8453, Marshal Scientific, Hampton, NH, USA) was used to obtain optical absorption spectra from the SWCNT films on glass substrates. A Hall effect measurement system (Ecopia HMS-5300, Ecopia Corporation, Anyang-city, Gyeonggi-do, Republic of Korea) was employed to measure the carrier concentration, Hall coefficient, sheet resistance, and carrier mobility of the synthesized SWCNT films at a temperature of T = 300 K and a magnetic field of B = 0.518 T. The Cary 670 FTIR Spectrometer(Agilent Technologies, Miami, FL, USA) was used to identify the functional groups in the SWCNT thin films.

## 3. Results and Discussions

**Figure 2:**
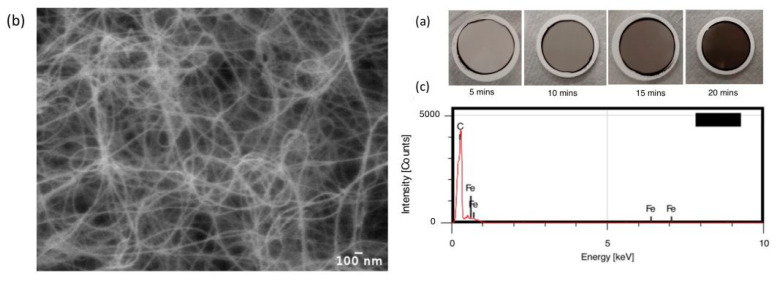
(**a**) Optical images of the samples with collection times of 5, 10, 15, and 20 min. (**b**) SEM image of SWCNT thin films. (**c**) EDS of the deposited SWCNTs.

SWCNTs were synthesized using the FCCVD method, with ferrocene (C_10_H_10_Fe) as the catalyst precursor, thiophene (C_4_H_4_S) as the growth promoter, ethanol (C_2_H_5_OH) as the carbon source, and a mixture of argon (Ar) and hydrogen (H_2_) as the carrier gas, with a total flow rate of 200 sccm. The growth temperature and the growth time were 1000 °C and 30 min, respectively. Figure 2a shows the optical images of samples with collection times of 5, 10, 15, and 20 min. As the collection time increases, the color of the deposited film changes from light gray to dark brown because of the increase in their thickness. Figure 2b illustrates a typical SEM image of SWCNT bundles that are connected and form a continuous 2D weblike structure [40,41]. Figure 2c shows that the deposited SWCNTs mainly contain C (carbon) and Fe (iron) elements. Fe is the catalyst nanoparticle coming from the catalyst precursor ferrocene and the carbon is from the SWCNTs.

**Figure 3:**
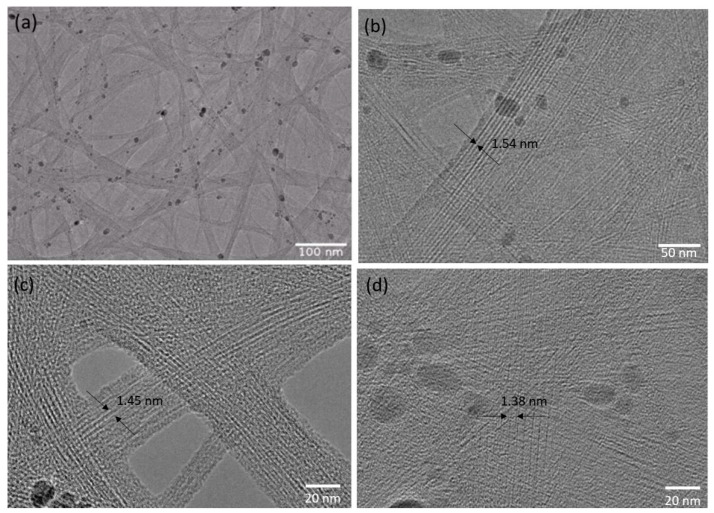
TEM images of deposited SWCNTs. (**a**) The overall morphology of the SWCNTs and bundles. (**b**–**d**) Different selected areas of the deposited film with the estimated average diameter of SWCNTs in the bundles, namely 1.54 nm, 1.45 nm, and 1.38 nm, respectively.

Figure 3 shows the TEM images of SWCNT bundles. The morphology of the network structure of the SWCNTs and bundles can be seen in Figure 3a. Figure 3b–d are high-resolution TEM images of the SWCNTs. Using ImageJ software (free online version 1.53t), the average diameters of the SWCNTs in the bundles for the selected areas were measured as 1.54 nm, 1.45 nm, and 1.38 nm, respectively. The average diameter is 1.46 nm, comparable with the reported values for the SWCNTs’ diameters, which were between 1 and 2 nm [42,43,44]. Hussain et al. [42] reported that the diameters of their SWCNTs synthesized by the FCCVD method were in the range of 1.3–1.5 nm. The diameter of the synthesized SWCNTs using the arc-discharge method was reported to be between 1.3 and 1.6 nm [45].

**Figure 4:**
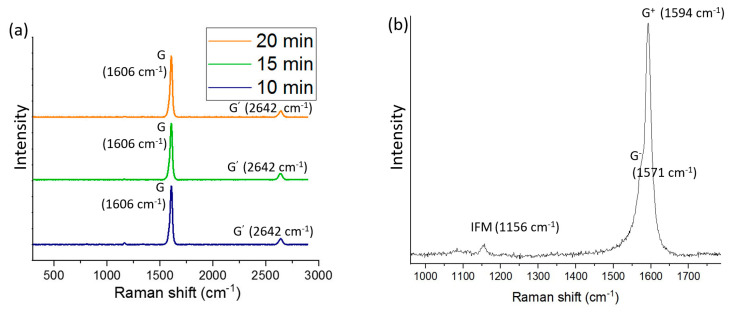
(**a**) Raman spectroscopy of the three samples with collection times of 10, 15, and 20 min using a 633.8 nm He-Ne Laser. (**b**) A zoomed image of the sample with a 20 min collection time using a 514 nm argon ion laser.

Figure 4a shows the Raman spectra for the three samples with collection times of 10, 15, and 20 min when employing a 633.8 nm He-Ne Laser, and Figure 4b shows a zoomed image for the Raman spectra of the sample with a collection time of 20 min when using a 514 nm argon ion laser. No peak appeared in the range of the D band when using the 633.8 nm He-Ne and 514 nm argon ion lasers. The emergence of a D band (usually around 1350 cm^−1^) is related to the defects of crystalline sp^2^ carbon structures [46,47]. Raman spectroscopy on graphene structures reveals that the D mode origin is from the edge defects [48]. The edge area in the well-crystallized SWCNTs is trivial because of the higher aspect ratio (>1000) and their atomic thickness. Therefore, they should not reveal the D band [49].

The G and G’ bands at 1606 cm^−1^ and 2642 cm^−1^, respectively, (Figure 4a), can be compared to the previously published values of 1591 cm^−1^ and 2659 cm^−1^ for the HiPCO SWCNTs (514 nm, Ar+ ion laser) [50]. The reason for the difference in the wavenumber can be attributed to the effect of the laser wavelength. The phonon frequencies of Raman signals can vary according to the change in the laser excitation energy [51]. The G band specified for all the sp^2^ carbon materials is related to the in-plane bond stretching mode of the C-C bonds in the hexagonal lattice [52]. The G’ (or 2D) band is a peak in the spectra of most sp^2^ carbon materials. The origin of this peak can be ascribed to a vibrational mode that was identified by the breathing of six carbons related to a hexagon in the graphene lattice. When increasing the collection time, no change was observed in the position of the peaks (Figure 4a). Because of the electron–phonon coupling or strain effect in SWCNTs, the G band splits into G^+^ and G^−^ peaks. The split between the G^+^ (at 1594 cm^−1^) and G^−^ (at 1571 cm^−1^) peaks can be seen in Figure 4b. The G^−^ peak is usually unseparated from the G^+^ peak; however, it can be varied in shape [53]. The signal related to the G^−^ peak is attributed to the curvature of SWCNTs (i.e., diameter), which is specific to their electronic characteristics [54]. A small peak that appeared at 1156 cm^−1^ (Figure 4b) is related to the intermediate frequency modes (IFM mode). These modes, which are usually reported around the range of 600–1200 cm^−1^, are considered weak and insignificant features; they exist in all graphene-related materials [36] and are assigned to second-order, two-phonon, or one-phonon and one-elastic scattering double resonance Raman processes [55,56].

**Figure 5:**
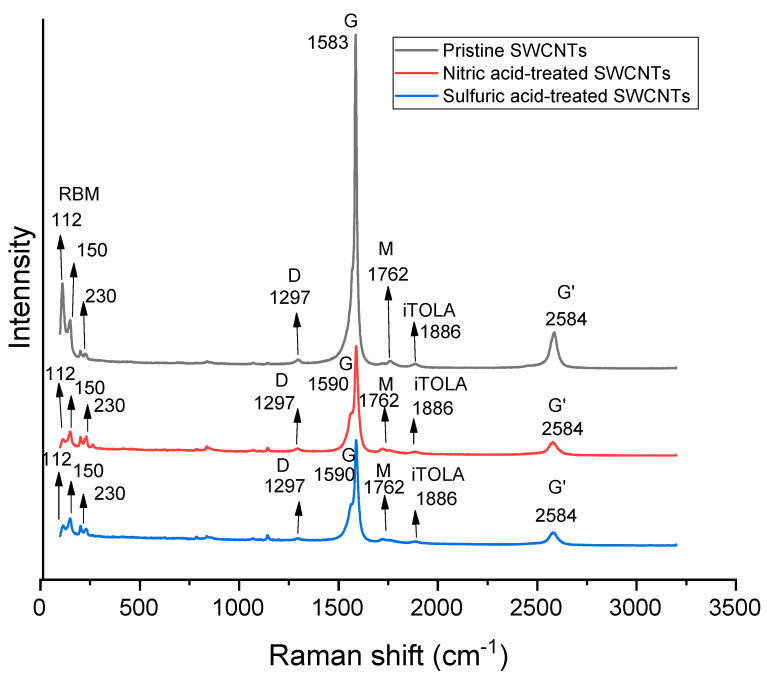
Raman spectra of the pristine SWCNTs, nitric-acid-treated SWCNTs, and sulfuric-acid-treated SWCNTs using a 785 nm diode laser.

Figure 5 shows the Raman spectra of the pristine SWCNTs, nitric-acid-treated SWCNTs, and sulfuric-acid-treated SWCNTs using a 785 nm diode laser. Using this laser, a weak peak was observed at 1297 cm^−1^ for all the samples that can be related to the D mode. The obtained I_D_/I_G_ values were 0.11, 0.22, and 0.14 for the pristine SWCNTs, nitric acid-treated SWCNTs, and sulfuric-acid-treated SWCNTs, respectively. The low I_D_/I_G_ values confirm that our synthesized SWCNTs are very well crystallized and have a high quality. The higher I_D_/I_G_ values for the acid-treated samples show that the acid treatment decreased the amount of graphenic sp^2^-carbons in the pristine SWCNTs. The I_D_/I_G_ values we obtained can be compared to the values of 0.2 and 0.37 achieved by Wang et al. [57] for their pristine SWCNTs and sulfuric-acid-treated SWCNTs, respectively. An up-shift related to the G-frequency was observed after the acid treatments. This phenomenon was also detected by Tantang et al. [58] after the acid treatment. They related the change in the shift to the possibility of an increase in charge carrier-accepting defects after the acid treatment. The peak at 1762 cm^−1^ can be related to the overtone of the out-of-plane infrared active mode in graphite, which is called the M band. The peak at 1886 cm^−1^ can be assigned to the iTOLA band originating from a combination of a phonon from the in-plane transverse optical (iTO) or longitudinal optical (LO) branch and a longitudinal acoustic (LA) branch phonon [59]. The peaks at 112, 150, and 230 are related to the radial breathing modes (RBMs).

The structural characteristics of our synthesized SWCNTs showed the morphology of the SWCNTs in bundles, with an average diameter of 1.46 nm. The deposited SWCNTs mainly contain carbon and iron elements. The low value of I_D_/I_G_ (0.11) for the pristine SWCNTs in the Raman spectroscopy measurements indicates that our synthesized SWCNTs are very well crystallized and have a high quality. This ratio increased to 0.14 and 0.22 after the treatment with sulfuric acid and nitric acid, respectively.

We carried out the Hall effect measurements on the SWCNT films with different collection times (5, 10, 15, 20, and 25 min). Important electrical properties such as the carrier concentration (carrier density), mobility, Hall coefficient, and sheet resistance of the SWCNT films were explored through these measurements. The magnitudes of the carrier concentration were observed to be between 4.6 × 10^21^ and 1.3 × 10^23^ cm^−3^. These values are comparable to the carrier density of 10^21^–10^22^ cm^−3^ obtained for the purified SWCNTs synthesized through a high-pressure CO conversion process (HiPCO CNTs) or laser ablation method (LA CNTs) and chemically treated with SOCl_2_ [36]. Compared to the values of 10^18^–10^19^ cm^−3^, which were earlier reported for the CNT films [60,61,62] and bundles [63], our results are closer to the theoretically predicted value of ~10^22^ cm^−3^ that was calculated for the aligned metallic CNTs [37]. The higher carrier density of our SWCNTs can be attributed to the advances in the synthesis method used for SWCNT films and their purity [36]. For example, the dry FCCVD method allows for the production of films with longer CNTs and exceptional optoelectronic properties by resolving the tradeoff between the CNT length and solubility during film fabrication [20]. The mobility of the synthesized SWCNTs was in the same order of magnitude for all the collection times, with an average value of ~0.034 cm^2^/Vs. This mobility is very close to the one observed for pristine HiPCO SWCNTs (0.04 cm^2^/Vs) and reported by Lee et al. [36]. However, these Hall mobilities were much lower than the values for the field-effect mobility, namely 220 and 100,000 cm^2^/Vs, reported for the individual semiconducting SWCNTs. The factors limiting the Hall mobility were thought to be random networks of CNTs and barriers at the inter-tube junctions of the CNT films [36,64,65].

**Figure 6:**
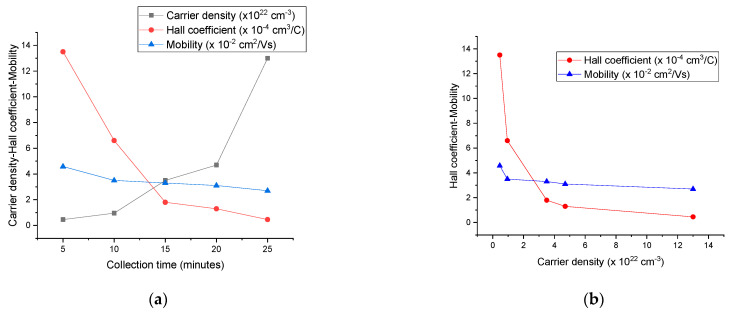
Graphs of (**a**) carrier density, Hall coefficient, and mobility versus collection time of SWCNT films, and (**b**) Hall coefficient and mobility versus carrier density.

In Figure 6a, the carrier concentration, Hall coefficient, and mobility are plotted versus the collection time of the SWCNT films. The carrier concentration increased from 4.6 × 10^21^ to 1.3 × 10^23^ cm^−3^ by increasing the collection time from 5 to 25 min, while the Hall coefficient decreased from 1.35 × 10^−3^ to 4.6 × 10^−5^ (also see Table 1). However, the decrease in mobility was insignificant. Equation (1) shows that an increase in the carrier concentration will lead to a decrease in the Hall coefficient, as we can see in Figure 6b. Also, when increasing the carrier concentration, a slight decrease was observed in the mobility of the SWCNT thin films (Figure 6b). After reaching a certain thickness value (around 800 nm for 25 min of collection time), the further increase did not significantly change the carrier concentration of our thin films, meaning that the carrier concentration became independent of the thickness. The Hall effect measurements for the samples with collection times of 5, 10, 15, 20, and 25 min are summarized in Table 1. The sign of the Hall coefficients was positive for all samples, showing that the synthesized SWCNTs are p-type materials.

R_H_ = 1/*n*e (1)

where R_H_ is the Hall coefficient (cm^3^/C), *n* is the carrier concentration (cm^−3^), and e = 1.6 × 10^−19^ (C).

**Figure 7:**
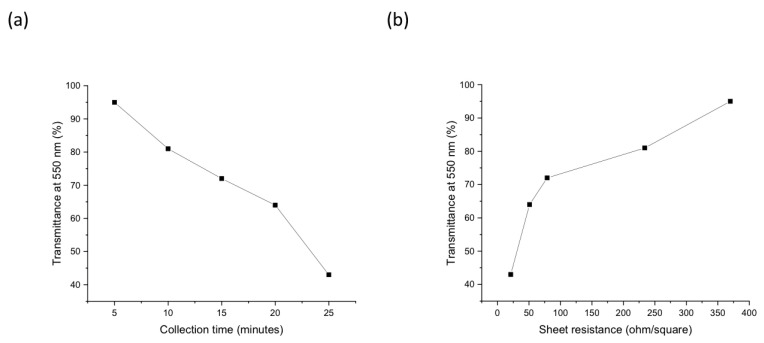
Graphs of the transmittance versus (**a**) collection time and (**b**) sheet resistance.

Figure 7a,b show the transmittance versus the collection time and sheet resistance. When increasing the collection time from 5 to 25 min, the transmittance and sheet resistance of the samples decreased from 95% to 43% and from 370 to 21 ohm/square, respectively. The best result for the sheet resistance and transmittance (T) achieved for the pristine Arc SWCNT TCFs was reported to be 390 ohm/square at 90% T for 550 nm light [66]. This can be compared to our SWCNT films with a sheet resistance of 370 ohm/square at 95% T. Increasing the collection time leads to an increase in the thickness of the thin films, which leads to a decrease in the sheet resistance of the materials, as indicated by Equation (2). The observed sheet resistance and transmittance magnitudes are comparable with the ones reported for the SWCNT thin films [35,39,42].

R_s_ = *p*/t (2)

where R_s_ is the sheet resistance (ohm/square), *p* is the resistivity (ohm·cm), and t is the thickness of the thin film (cm).

Important electrical characteristics of the SWCNTs, including the carrier mobility, carrier density, Hall coefficient, and sheet resistance, were obtained using Hall effect measurements. The positive signs of the Hall coefficients showed that the majority of the carriers were holes in our synthesized SWCNT thin films. The samples with a transmittance between 95 and 43% showed a high carrier density of 10^21^–10^23^ cm^−3^, with a sheet resistance between 370 and 21 ohm/square. 

Different acid treatments were performed on the SWCNT films using the same collection time to investigate the effect of doping on their electrical properties. The conclusion obtained from treating one sample can be applied to the samples with different collection times. The samples with a 10 min collection time (transmittance of 80%) were treated with sulfuric, nitric, and hydrochloric acid at different acid concentrations (Table 2). All the samples were rinsed with deionized (DI) water after being treated with the acids; meanwhile, in some cases (Case C in Table 2), the samples were first rinsed with ethanol followed by DI water. In Case B, the samples were heated to 80 °C throughout the duration of the treatment (30 min). Hall effect measurements were applied to the samples after the treatments to study the change in their electrical characteristics.

All the samples maintained their p-type characteristic after being treated with the acids. Acceptor-type doping of the SWCNTs was observed by other researchers using Brønsted acids (like sulfuric, nitric, and hydrochloric acid) [29,66,67,68]. Compared to the sample without any treatment (Case G), the hole mobility increased in all acid-treated samples, the hole concentration of all sulfuric acid and hydrochloric-acid-treated samples decreased, but the hole concentration of the nitric-acid-treated sample increased. The experimental results showed that nitric acid treatment can improve the SWCNT film’s mobility and carrier concentration (Case F). The corresponding conductivity was calculated for the samples using Equation (3).

σ = μne(3)

Here, σ is the conductivity due to holes in a unit of Siemens per meter (S/m) or Ω^−1^m^−1^, n and e stand for the electronic carrier concentration and electron charge, respectively, and μ is the hole mobility.

Despite a slight decrease in the hole concentration, the conductivity increased in all kinds of acid treatments while the sheet resistance decreased, as can be seen in Table 2. Although the sample treated with sulfuric acid at 80 °C (Case B) showed the highest hole mobility, the highest conductivity (30,720 S/m) was associated with the nitric acid treatment (Case F). Additionally, there was a significant decrease in the sheet resistance of the nitric-acid-treated sample (Case F), which decreased to 43 ohm/square, compared to the untreated sample, which had a sheet resistance of 243 ohm/square (Case G). This value of sheet resistance (43 ohm/square) is lower than the value of 59 ohm/square at 80% T that was achieved for the HNO_3_-doped SWCNT films (synthesized via arc-discharge method) reported by Paul et al. [69]. The second and third highest conductivity are 14,060 S/m and 12,096 S/m for the films treated with hydrochloric acid and DI water rinsing (Case E) and those treated with the sulfuric acid treatment, with ethanol as well as DI water rinsing (Case C), respectively. All the calculated conductivities were in the range reported (in the order of 10^2^ to 10^6^ S/cm) for SWCNT [70]. The improvement in the conductivity of the SWCNT films can be related to the downshifts in the Fermi level toward the valence bands of the SWCNTs, leading to a reduction in the Schottky barrier height and increasing the conductivity of the films [35,71].

**Figure 8:**
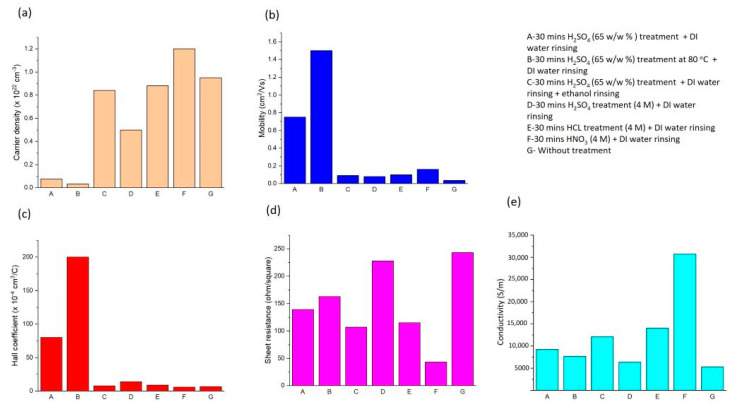
Bar graphs showing the effect of different acid treatments on the (**a**) carrier density, (**b**) hole mobility, (**c**) Hall coefficient, (**d**) sheet resistance, and (**e**) conductivity of the SWCNT thin films.

The bar graphs (Figure 8 ) show the comparison between the electrical properties of the SWCNT thin films under different acid treatments according to the data in Table 2. Overall, we can see the enhancement in the electrical properties of the SWCNT films after the acid treatments, with an increase in the hole mobility and conductivity, as well as a decrease in the sheet resistance in all cases. Out of all the samples, the ones treated with sulfuric acid at a temperature of 80 °C (Case B) showed the greatest hole mobility, at 1.5 cm^2^/Vs (Figure 8b). However, the sample treated with nitric acid (Case F) demonstrated the highest carrier conductivity, at 30,720 S/m (Figure 8e), the highest carrier density, at 1.2 × 10^22^ cm^−3^ (Figure 8a), and the lowest sheet resistance, at 43 ohm/square (Figure 8d).

**Figure 9:**
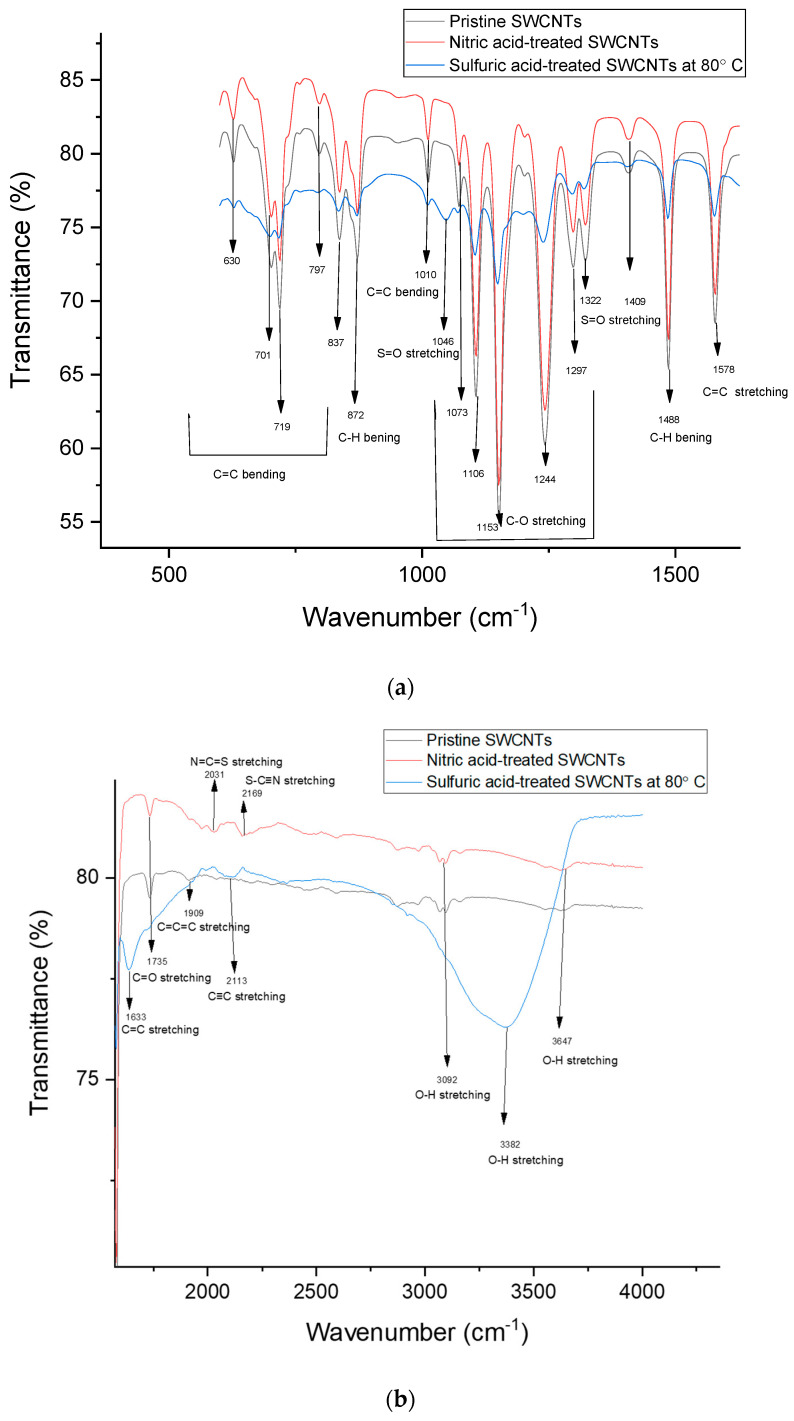
FTIR spectra of pristine SWCNTs, HNO_3_-treated SWCNTs, and H_2_SO_4_-treated SWCNTs heated at 80 °C in the wavelength ranges of (**a**) 500–1600 cm^−1^ and (**b**) 1600–4000 cm^−1^.

Fourier-Transform Infrared Spectroscopy (FTIR) was utilized to analyze the chemical structure of the sulfuric-acid-treated SWCNTs at 80 °C (Case B) and the nitric-acid-treated SWCNTs (Case F) in comparison to the pristine SWCNTs (Case G). The analysis was conducted by identifying functional groups and comparing any of the changes observed in the three cases. Figure 9 shows the FTIR of the pristine SWCNTs (Case G), HNO_3_-treated SWCNTs (Case F), and H_2_SO_4_-treated SWCNTs heated at 80 °C (Case B) in the wavelength ranges of 500–1600 cm^−1^ (Figure 9a) and 1600–4000 cm^−1^ (Figure 9b). The peaks at 630, 701, 719, 797, 837, and 1010 cm^−1^ in all three samples are related to the C=C bending of the alkene functional group [72,73], while the peak at 872 cm^−1^ comes from the bending of the C-H for the benzene derivative functional group [74]. Hydrogen exists in the precursor solution for the synthesis of SWCNTs and is also used as a carrier gas during the synthesis process. A new peak appeared at 1046 cm^−1^ for the sulfuric-acid-treated sample, which represents the S=O stretching of sulfoxide [75] and is attributed to the sulfuric acid treatment. The peaks at 1073, 1106, 1153, 1244, and 1297 cm^−1^, common in all three samples, are related to the C-O stretching mode of the primary alcohol, secondary alcohol, tertiary alcohol, and alkyl aryl ether functional groups [72,76]. Ethanol is a primary alcohol that is used in the precursor solution. Secondary and tertiary alcohols can form by attaching the hydroxyl group (-OH) to a carbon atom that is bonded to two and three alkyl groups (or hydrocarbon chains), respectively [77]. Ether is formed when an oxygen atom is attached to two alkyl or aryl groups (aromatic hydrocarbons) [78]. For all samples, the peak at 1322 cm^−1^ represents the S=O stretching mode of the sulfone, and the peak at 1409 cm^−1^ is related to the S=O stretching mode of the sulfate functional group [72,79]. Sulfur comes from thiophene, which is used in the precursor solution.

The 1488 and 1578 cm^−1^ peaks appearing in all three samples originate from the C-H bending of alkane and the C=C stretching of cyclic alkane functional groups, respectively [80]. The peak at 1735 cm^−1^ for pristine and nitric-acid-treated SWCNTs corresponds to the C=O stretching mode in the carbonyl or carboxylic acid groups [81]. This peak (1735 cm^−1^) is downshifted to 1633 cm^−1^ for the sulfuric-acid-treated sample, representing the C=C stretching of alkene [82]. The sulfuric-acid-treated sample also shows a new peak at 2113 cm^−1^, which is related to the C≡C stretching of the alkyne functional group. This peak can be compared to the one at 1909 cm^−1^ for the pristine SWCNT sample showing the C=C=C stretching of the allene functional group [72]. This change in the bond formation, from double-bond carbon in the pristine SWCNTs into triple-bond carbon in the sulfuric-acid-treated sample, can be attributed to the heating performed during the acid treatment. Wong et al. [83] conducted simulations on the distribution of chemical bonds in carbon chains. Their findings suggest that as the temperature rises, the carbon atoms in a compound may transition from a double bond to a single or triple bond. This transition is dependent on energy minimization and the Octet rule. The Octet rule dictates that an atom’s valence shell can only accommodate a maximum of eight electrons, with no lone pair electrons are permitted.

In the range of 1600–4000 cm^−1^, two new peaks appeared at 2031 and 2169 cm^−1^ for the nitric-acid-treated sample. These peaks can be related to the N=C=S stretching of isothiocyanate and the S-C≡N stretching of thiocyanate, respectively [72]. Sulfur exists in the precursor solution created by thiophene. The peaks at 3092 and 3647 cm^−1^ appeared for the pristine and nitric-acid-treated SWCNTs, corresponding to the O-H stretching of alcohol (hydroxyl functional group). The peak at 3382 cm^−1^ for the sulfuric-acid-treated sample is also related to the O-H stretching of alcohol [84]. These peaks are usually broad and strong in the 3200–3550 cm^−1^ range [72], as seen in Figure 9b. The disappearance of one of the peaks related to O-H stretching and also the peak related to C=O stretching (at 1735 cm^−1^) for the sulfuric-acid-treated sample might be a reason for the increase in the hole mobility of this sample. Studies have shown that polar groups such as hydroxyl and carbonyl functional groups can form deep traps that can capture electric charge carriers [85,86]. The scattering and capture of free charges by deep traps can lead to energy loss in the charge carriers and a decrease in their mobility [86]. Therefore, the reduction in the number of polar groups such as carbonyl and hydroxyl functional groups in the sulfuric-acid-treated sample might play a role in improving its mobility. Compared to the sulfuric-acid-treated sample, the FTIR spectra of the nitric-acid-treated sample are more similar to the pristine SWCNTs’ spectra. However, the improved electrical properties of the nitric-acid-treated SWCNTs might be related to the existence of two new peaks (2031 and 2169 cm^−1^) in this sample, representing the isothiocyanate and thiocyanate functional groups. Polymers containing thiocyanate have been widely reported for use as hole-transporting layers in optoelectronic applications (such as solar cells) due to the improvement in the charge extraction efficiency associated with increased conductivity [87,88].

In summary, among the different acid treatments, the sample treated with sulfuric acid at a temperature of 80 °C (Case B) exhibited the highest hole mobility value, at 1.5 cm^2^/Vs. In addition, the sample treated with nitric acid (Case F) demonstrated the highest carrier conductivity, at 30,720 S/m, the highest carrier density, at 1.2 × 10^22^ cm^−3^, and the lowest sheet resistance, at 43 ohm/square. These improvements in the electrical properties of both samples make them suitable for use in electronic applications such as thin film transistors or solar cells.

## 4. Conclusions

SWCNT films were synthesized through a floating catalyst chemical vapor deposition method using a total low flow rate of 200 sccm for the mixed argon and hydrogen gases. The structural properties of the thin films were investigated by SEM, TEM, FTIR, and Raman spectroscopy. The low value of I_D_/I_G_ (0.11) in the Raman spectroscopy measurements of our pristine synthesized SWCNTs confirms that the SWCNT films are highly crystallized. This value increased to 0.14 and 0.22 after the treatment with sulfuric acid and nitric acid, respectively. The significant electrical characteristics of the SWCNT films, such as the carrier concentration, carrier mobility, Hall coefficient, and sheet resistance, were investigated through Hall effect measurements. The samples showed a high carrier concentration of 10^21^–10^23^ cm^−3^, with a transmittance between 95 and 43%. The effect of different acid treatments was explored using nitric, sulfuric, and hydrochloric acids. In all the treatments, an increase was observed in their carrier mobility and conductivity, while their sheet resistance decreased. The samples retained their p-type nature after the acid treatments, showing that the treatments led to p doping. The samples treated with sulfuric acid at 80 °C showed the highest mobility, at 1.5 cm^2^/Vs. On the other hand, the highest carrier conductivity (30,720 S/m) and the lowest sheet resistance (43 ohm/square) were obtained from the samples treated with nitric acid. The presence of multiple functional groups in our synthesized SWCNTs may increase their potential for various applications, since each functional group exhibits distinct characteristics and reactivity, enabling participation in various types of reactions and increasing functionality. Improving the electrical properties of SWCNTs can lead to a great enhancement in their electronic applications. Since the synthesized SWCNT films maintained their p-type nature after the acid treatments, the enhancement in their electrical characteristics makes them suitable candidates for hole-transporting layers in solar cells.

## Figures and Tables

**Table 1:** Hall effect measurements for the samples with collection times of 5, 10, 15, 20, and 25 min.

Carrier Density (cm^−3^ )	Mobility (cm^2^/Vs)	Hall Coefficient (cm^3^/C)	Carrier Type	Sheet Resistance (ohm/Square)	Collection Time (Minutes)
4.6 × 10^21^	4.58 × 10^−2^	1.35 × 10^−3^	P-type	370	5
9.5 × 10^21^	3.5 × 10^−2^	6.6 × 10^−4^	P-type	234	10
3.5 × 10^22^	3.3 × 10^−2^	1.8 × 10^−4^	P-type	79	15
4.7 × 10^22^	3.1 × 10^−2^	1.3 × 10^−4^	P-type	51	20
1.3 × 10^23^	2.7 × 10^−2^	4.6 × 10^−5^	P-type	21	25

**Table 2:** Hall effect measurements of the SWCNT film of collection time of 10 min after acid treatments.

Case	Acid Treatment	Carrier Density (cm^−3^)	Hole Mobility (cm^2^/Vs)	Hall Coefficient (cm^3^/C)	Carrier Type	Sheet Resistance (ohm/Square)	Conductivity (S/m)
A	30 min H_2_SO_4_ (65 *w*/*w* %) treatment + DI water rinsing	7.7 × 10^20^	0.75	8 × 10^−3^	P-type	139	9240
B	30 min H_2_SO_4_ (65 *w*/*w* %) treatment at 80 °C + DI water rinsing	3.2 × 10^20^	1.5	2 × 0^−2^	P-type	163	7680
C	30 min H_2_SO_4_ (65 *w*/*w* %) treatment + DI water rinsing + ethanol rinsing	8.4 × 10^21^	0.09	7.7 × 10^−4^	P-type	107	12,096
D	30 min H_2_SO_4_ treatment (4 M) + DI water rinsing	5 × 10^21^	0.08	1.4 × 10^−3^	P-type	228	6400
E	30 min HCL treatment (4 M) + DI water rinsing	8.8 × 10^21^	0.1	8.8 × 10^−4^	P-type	115	14,060
F	30 min HNO_3_ (4 M) + DI water rinsing	1.2 × 10^22^	0.16	5.8 × 10^−4^	P-type	43	30,720
G	Without treatment	9.5 × 10^21^	0.035	6.6 × 10^−4^	P-type	243	5320

## Data Availability

The raw data required to reproduce these findings are available upon request from the corresponding author. The processed data required to reproduce these findings are available upon request from the corresponding author.
